# Distribution of silver in rats following 28 days of repeated oral exposure to silver nanoparticles or silver acetate

**DOI:** 10.1186/1743-8977-8-18

**Published:** 2011-06-01

**Authors:** Katrin Loeschner, Niels Hadrup, Klaus Qvortrup, Agnete Larsen, Xueyun Gao, Ulla Vogel, Alicja Mortensen, Henrik Rye Lam, Erik H Larsen

**Affiliations:** 1 Division of Food Chemistry, National Food Institute, Technical University of Denmark, Søborg, Denmark; 2 Division of Toxicology and Risk Assessment, National Food Institute, Technical University of Denmark, Søborg, Denmark; 3 Department of Biomedical Sciences, The Panum Institute, University of Copenhagen, Copenhagen N, Denmark; 4 Department of Neurobiology, Institute of Anatomy, University of Aarhus, Aarhus C, Denmark; 5 Lab for Bio-Environmental Effects of Nanomaterials and Nanosafety, Institute of High Energy Physics, Chinese Academy of Sciences, Beijing, China; 6 The Nanotoxicology and Occupational Hygiene Group, National Research Centre for the Working Environment, Copenhagen Ø, Denmark; 7 Human Health and Safety, DHI, Hørsholm, Denmark

## Abstract

**Background:**

The study investigated the distribution of silver after 28 days repeated oral administration of silver nanoparticles (AgNPs) and silver acetate (AgAc) to rats. Oral administration is a relevant route of exposure because of the use of silver nanoparticles in products related to food and food contact materials.

**Results:**

AgNPs were synthesized with a size distribution of 14 ± 4 nm in diameter (90% of the nanoparticle volume) and stabilized in aqueous suspension by the polymer polyvinylpyrrolidone (PVP). The AgNPs remained stable throughout the duration of the 28-day oral toxicity study in rats. The organ distribution pattern of silver following administration of AgNPs and AgAc was similar. However the absolute silver concentrations in tissues were lower following oral exposure to AgNPs. This was in agreement with an indication of a higher fecal excretion following administration of AgNPs. Besides the intestinal system, the largest silver concentrations were detected in the liver and kidneys. Silver was also found in the lungs and brain. Autometallographic (AMG) staining revealed a similar cellular localization of silver in ileum, liver, and kidney tissue in rats exposed to AgNPs or AgAc.

Using transmission electron microscopy (TEM), nanosized granules were detected in the ileum of animals exposed to AgNPs or AgAc and were mainly located in the basal lamina of the ileal epithelium and in lysosomes of macrophages within the lamina propria. Using energy dispersive x-ray spectroscopy it was shown that the granules in lysosomes consisted of silver, selenium, and sulfur for both AgNP and AgAc exposed rats. The diameter of the deposited granules was in the same size range as that of the administered AgNPs. No silver granules were detected by TEM in the liver.

**Conclusions:**

The results of the present study demonstrate that the organ distribution of silver was similar when AgNPs or AgAc were administered orally to rats. The presence of silver granules containing selenium and sulfur in the intestinal wall of rats exposed to either of the silver forms suggests a common mechanism of their formation. Additional studies however, are needed to gain further insight into the underlying mechanisms of the granule formation, and to clarify whether AgNPs dissolve in the gastrointestinal system and/or become absorbed and translocate as intact nanoparticles to organs and tissues.

## Background

Silver nanoparticles (AgNPs) are presently one of the most frequently used nanomaterials in consumer products [1] because of their proposed antimicrobial properties [2]. Silver in the form of Ag^+ ^ions has toxic effects on many pathogens, including bacteria, viruses, and fungi [3]. Because of its relatively low toxicity in humans, silver has been used in various medical applications [4]. The antibacterial activity of AgNPs also depends on the Ag^+ ^ion [3], which is readily formed on the nanoparticle surface due to reaction with oxygen. The antibacterial activity of AgNPs increases with decreasing particle size which has been associated with the increasing surface area-to-mass ratio [3].

In general, application of nanoparticles in products related to food and beverages is still rare [5]. AgNPs however, can already be found in a number of commercial products including food packing materials and kitchen appliances, and is even sold as an alternative "health supplement" [5,6]. Furthermore, silver nanoparticles are considered as a potential additive to animal feed to replace antibiotics [7,8]. Therefore, oral intake of silver nanoparticles is a relevant route of exposure for the consumer.

The toxicology of silver and its compounds has been studied for decades. Animal studies dealing with oral exposure to silver are nevertheless scarce. A summary of the available data has been given in the "Toxicological Profile for Silver" from the Agency for Toxic Substances and Disease Registry of the U.S. Public Health Service [9]. Although the toxicology of silver and its compounds has been studied, several knowledge gaps exist concerning the risk caused by silver in the form of nanoparticles, both to humans and to the environment [10]. It is for example not known to what extent intact AgNPs themselves enter the body or whether only silver ions originating from the nanoparticles are absorbed.

It is generally known that nanoparticles can be taken up by the intestinal system not only via M-cells in the Peyer's patches and the isolated follicles of the intestinal-associated lymphoid tissue, but also via enterocytes [11]. The degree of absorption is mainly governed by nanoparticle size, surface charge, hydrophobicity, and the presence or absence of surface ligands. It is generally agreed that absorption increases with decreasing particle diameter for sizes below 1 μm [12]. Thus, aggregation of nanoparticles in the lumen of the gastro-intestinal tract can lead to decreased absorption.

In a 28-day repeated oral dose toxicity study, AgNPs of 60 nm in diameter and stabilized by 0.5% aqueous carboxymethylcellulose, were administered to rats [13]. A dose-dependent increase of the silver concentration in the rat organs was observed with the highest concentrations in stomach, kidney and liver. However, it was not investigated whether the silver reached the tissues and organs as nanoparticles. Animal studies using other exposure routes revealed that intact AgNPs indeed could translocate to and deposit in a variety of organs of experimental animals. Following subcutaneous injection of 50-100 nm diameter AgNPs, discrete nanoparticles were found in kidney, liver, spleen, and lung tissue, as well as in vascular endothelial cells of the blood-brain barrier [14]. Intraperitoneally injected bovine serum albumin (BSA)-coated AgNPs of 2 nm in diameter were detected as larger aggregates in liver, kidney, and heart tissue, which was attributed to coalescence or aggregation events taking place in the blood serum or in the tissue [15].

The aim of the present study was to investigate the organ distribution and cellular localization of silver in rats following 28 days repeated oral exposure of either AgNPs or silver acetate (AgAc). AgNPs were sterically stabilized with a polymer to assure the availability of well-characterized and stable nanoparticles throughout the duration of the animal experiment. AgAc was chosen for comparison because it is a soluble salt containing a biocompatible counter ion. Transmission electron microscopy (TEM) was applied to investigate whether intact AgNPs entered the intestinal wall of rats.

## Methods

Unless stated otherwise, results based on repeated measurements are given as mean ± 1s.d. The number of repetitions N is stated in parentheses.

### Synthesis of the AgNPs

AgNPs were produced by reducing silver nitrate with hydrazine in the presence of polyvinylpyrrolidone (PVP) [16]. An amount of 300 mg of PVP powder (PVP-K30, BASF Ludwigshafen, Germany) was dissolved in 8 ml of an aqueous 25 mM silver nitrate solution (AgNO_3_, A.R Beijing Chemical Factory, China). The mixture was diluted with 14 ml deionized water. After 5 min at room temperature 4 ml of 70 mM hydrazine (N_2_H_4_·H_2_0, A.R Beijing Zhonglian Reagent Fine Chemicals Co., Ltd., China) was added dropwise. Hydrazine was added in stoichiometric excess to achieve complete reduction of the silver salt. All steps were performed under continuous stirring. The final suspension was purified by centrifugation at 4,000 rpm (1,467 × *g*) for 10 min and by dialysis for 24 h against deionized water using a 14 kDa membrane. The nanoparticle suspension was prepared in several batches which were later combined. Each batch was controlled by dynamic light scattering to ensure a similar size distribution of the AgNPs in each batch. An average nominal silver concentration of 450 μg/ml was estimated after production of the AgNPs and was based on measurements by inductively coupled plasma optical emission spectrometry.

### Characterization of the nanoparticle suspension

The silver concentration of the AgNP suspensions was determined by using a quadrupole-based inductively coupled plasma mass spectrometer (ICP-MS 7500ce, Agilent Technologies, Japan). Before measurement, the suspensions were digested in 1 ml concentrated nitric acid (PlasmaPure, SCP Science, Quebec, Canada) in a microwave oven (Multiwave, Anton Paar, Graz, Austria) at elevated temperature and pressure (~ 250°C, ~ 70 bar). From three different bottles, five to six subsamples of 40 μl were transferred to high pressure quartz vessels. After digestion the samples were diluted 500-times with deionized water. The concentration of Ag was quantified against an external calibration curve (Ag stock solution 1,000 μg/ml obtained from SCP Science, Quebec, Canada) with internal standardization (rhodium (Rh) stock solution 1000 μg/ml, SCP Science, Quebec, Canada). Prior to analysis the samples were further diluted (dilution factor 25), resulting in a nitric acid concentration of approximately 2% v/v.

The silver isotope ^107^Ag was chosen for the ICP-MS measurements because a slightly lower detection limit was obtained for this isotope. The limit of detection for the AgNP suspensions, which was estimated at 0.7 μg Ag/ml, was based on three times the standard deviation from repeated measurements of blank solutions.

To determine the amount of silver in the suspensions that was not present as nanoparticles, filtrates of three subsamples were prepared by centrifugation through a micro centrifuge cellulose acetate filter with a molecular weight cut-off of 12 kDa (VectaSpin Micro Centrifuge Filter, Whatman, England). A volume of 500 μl of 10-times diluted suspension was centrifuged at 11,000 rpm (8,117 × *g*) for 30 min in a micro centrifuge (Eppendorf MiniSpin, Eppendorf AG, Hamburg, Germany). Because the AgNPs and the polymer could clog the membrane before all unbound silver had passed the filter, the residue in the filter cartridge was re-suspended twice in 500 μl of deionized water and filtered again. In order to investigate whether any silver gradually dissolved from the AgNPs over time, silver concentrations were determined in the filtrates at 3 and 16 weeks before and 5 months after the animal study.

Before analysis of the silver concentration by ICP-MS the filtrates were diluted 100-times with 2% v/v nitric acid and Rh was added as internal standard (10 ng/ml). The limit of detection for silver in the filtrates was 0.002 μg/ml. To assure the quality of the previous results a Sorvall Discovery 90SE ultracentrifuge (Kendro Laboratory Products, Asheville, NC, USA) with an AH-629 swinging bucket rotor was used as an alternative method for separation of the nanoparticles from the original AgNP suspension. A 10-times diluted suspension was transferred to a 17 ml thin walled polyallomer tube and centrifugated at 29,000 rpm (135,293 × *g*) for 19 h at 20°C; 500 μl of supernatant were taken from the surface of the liquid. According to centrifugation theory the maximum particle diameter in the supernatant was below 1 nm [17]. The supernatant was analyzed by ICP-MS using dissolution in acid as before.

The particle size and shape were observed by TEM using a Philips CM100 instrument (FEI, Eindhoven, The Netherlands) operated at 80 kV accelerating voltage. The prepared silver suspensions were diluted 10-times with deionized water. A drop of the suspension was applied to a Formvar-coated copper grid.

Dynamic light scattering (DLS) and laser Doppler velocimetry for characterization of hydrodynamic size and zeta potential of the nanoparticles in solution were performed using a Zetasizer Nano-ZS instrument (Malvern Instruments, UK). Before measurements the samples were diluted 10-times with deionized water. For DLS 250 μl of suspension were transferred to a disposable low volume cuvette (Malvern, UK). After equilibration to a temperature of 25°C for 2 min, five measurements were performed using 12 runs of 10 s each. The laser power (attenuator index) and the measurement position within the cuvette were determined automatically by the instrument. For the calculation of the nanoparticle volume size distribution the viscosity of water was used. The influence of PVP on the viscosity was neglected because the PVP concentration in the 10-times diluted suspensions was only 0.1% w/v. The optical parameters for silver, namely index of refraction n = 0.1 and index of absorption k = 4.28 at 633 nm laser wavelength were applied according to Johnson and Christy [18]. The stability of the nanoparticles in suspension was monitored regularly by measuring the size distribution within 20 weeks after arrival of the suspensions until the beginning of as well as during the last week of the animal study.

For determination of the zeta potential, 800 μl of suspension was transferred to a clear disposable zeta cell (Malvern, UK). After equilibration to a temperature of 25°C for 2 min, five measurements were performed where the number of runs (10 to 100) was determined automatically by the instrument. The Smoluchowski approximation was used for calculation of the zeta potential.

### Animal study

Four week old female Wistar Hannover Galas rats with specific pathogen-free health status were purchased from M&B Taconic (Lille Skensved, Denmark). Upon arrival the rats were allowed to acclimatize for a week before the start of the experiment. The body weight of the animals at the beginning of the study was 107 ± 9 g (N = 28). The rats were housed conventionally two per cage (Macrolon, Techniplats Gazzada S. ar. L., Buguggiate, Italy) with a 12:12-h reversed light/dark cycle from 7 p.m. to 7 a.m. The room temperature was 22 ± 1°C and the relative humidity 55% ± 5%. In their cages, the rats were given *ad libitum *a standard diet (Altromin rat No.1324, Brogården, Gentofte, Denmark) and citric acid acidified tap water (to avoid microbiological contamination of drinking water). The animal study was performed under conditions approved by Danish Agency of Protection of Experimental Animals and the in-house Animal Welfare Committee.

The rats were randomly divided in three groups and received either an aqueous solution of 11.5 mg/ml PVP (vehicle control, N = 9), AgNPs (N = 9) or AgAc (N = 7) by oral gavage twice a day for 28 days. Additionally, one rat was included in each of the vehicle control, the AgNP and the AgAc groups, respectively, for tissue sampling for electron microscopy. The dosing volume was 10 ml/kg b.w. The aqueous solution of PVP was chosen as a vehicle control because the same solution was used as stabilizer for the AgNPs. PVP was also added to the AgAc solution (AgC_2_H_3_O_2_, CAS number 563-63-3, ReagentPlus, Sigma Aldrich, Denmark) to a concentration of 11.5 mg/ml. The AgAc solution remained clear and colorless throughout the duration of the study indicating that silver did not precipitate or become reduced. The daily dose of silver in the AgNP and AgAc group was 9.0 mg/kg b.w.

In week three of the 28-day study the rats were individually placed in metabolism cages for 24 h for the collection of urine and feces in tubes on dry ice. During this time the animals had free access to water but not to feed in order to eliminate the risk of contamination of urine and feces samples.

### Sample preparation

On day 29 of the study the rats fasted overnight, were anaesthetized by CO_2_/O_2_, and decapitated. Blood from the neck wound was collected on heparin and plasma was prepared by centrifugation (1000 × *g *at 0-4°C for 10 minutes) and necropsy was performed. The brain was removed, weighed, transferred to ice-cold 0.32 M sucrose and homogenized at 0-4°C. Samples from the stomach (part of corpus ventriculi), liver (lowest part of the left median lobe), kidney (part of the right kidney), lungs (right median lobe), and muscle (right biceps femoris) were taken from five to six animals per group and frozen for later ICP-MS analysis. Furthermore, samples from the liver, ileum, and kidney were fixed in 4% neutral buffered formalin for 24 hours and embedded in paraffin. For all rats samples were taken from the same parts of the organs, namely the lowest part of the left and right lateral lobe of the liver, the part of the ileum directly above the ileo-caecal junction and a cross section of the right kidney at the level of the renal papilla. Paraffin sections of 3 μm thickness were obtained by use of a microtome, collected on Superfrost plus slides (Menzel-Gläser, Germany), and oven dried overnight at 37°C.

### Determination of silver concentrations in tissue, blood plasma, urine, and feces by ICP-MS

Thawed subsamples of approximately 250 mg tissue (stomach, liver, kidney, lungs, and muscle) and approximately 200 μl of brain homogenate, urine, and blood plasma were analyzed for their silver concentration. Additionally, formalin-fixed tissue from the small intestine was analyzed. The mesenteric fat was dissected from the small intestine (sectioned between the pylorus and the caecum) and the was sample transversally divided in halves, which were analyzed separately. The whole amount of collected feces per animal was dried in an oven for 60 h at 80°C and homogenized. A dry matter content of 40 ± 4% w/w (N = 8) was determined by weighing the feces before and after the drying procedure; 10 mg of the dried feces were analyzed in duplicates. One sample was taken for analysis by scanning electron microscopy (SEM).

For assurance of the analytical accuracy the certified reference materials (CRM) Dogfish Liver DOLT-4 (NRC-CNRC, Canada) with a certified value for silver of 930 ± 70 ng/g was included in the analysis. The dry matter content of the reference material was 93.4% w/w. The average determined silver concentration was 997 ± 47 ng/g (N = 30) which did not deviate from the certified value. For feces samples the analytical accuracy was controlled by recovery of silver standard spiked to samples because no CRM with a certified silver concentration at the 100 μg/g concentration level was available. The recovery was 106 ± 2% (N = 3).

Deionized water and 0.32 M sucrose (for the brain homogenates) was analyzed as blank samples to ensure that no contamination by silver from these sources occurred during sample preparation.

All samples were digested using 3 ml concentrated nitric acid at elevated temperature and pressure (~ 250°C; ~ 70 bar) as described previously. Following digestion, 3 ml of concentrated hydrochloric acid (PlasmaPure, SCP Science, Quebec, Canada) were added. For formalin-fixed tissue, 5 ml of nitric and 5 ml hydrochloric acid (added after digestion) were used because of the larger sample size (around 600 mg). Preliminary studies (data not included) showed that incomplete recoveries were obtained for silver added to the CRM Bovine Liver 1577a (NIST, Gaithersburg, MD, USA) spiked at 4 μg/g if no HCl was added after digestion. The excess chloride facilitated the formation of soluble silver chloride complexes, thus preventing precipitation of insoluble AgCl by reaction with chloride naturally present in the biological material.

The digests were further diluted with 2% v/v nitric acid to a final acid concentration of 8-12% v/v. The measurements were performed as described above. The limit of detection was estimated at 0.7 to 2.0 ng/g in the biological samples and for feces at 200 ng/g.

The silver contents were compared using Student's two sample t-test (p < 0.05) after F-test of the equality of variances using an Excel spreadsheet (Microsoft Corp., WA, USA).

### Autometallography (AMG)

After deparaffinization of the sections with xylene and rehydration through a series of ethanol-water washes, the slides were placed in jars filled with AMG developer [19]. The jars were transferred to a 26°C water bath covered with a dark hood. After 60 min the AMG development was stopped by replacing the developer with a 5% w/v sodium thiosulphate solution and leaving it for 10 min. Next, the sections were rinsed in running water for several minutes followed by a rinse in distilled water. Finally, the sections were counterstained with 0.1% m/v toluidine blue solution, dehydrated in ethanol-water washes, and after a xylene wash were embedded in mounting medium and covered with glass cover slips. Sections of control animals were always included.

### Animals and sample preparation for transmission and scanning electron microscopy

The rats were pre-anaesthetised with inhalation of Halothane 3% (Halocarbon Laboratories, River Edge, NJ, USA). Anaesthesia was induced by doses of a 9:1 mixture of Ketamine 25 mg/ml (Pfizer, Ballerup, Denmark) 90 mg/kg b.w. and Xylazine 20 mg/ml (Rompun^®^Vet. BAYER, Leverkusen, Germany) 10 mg/kg b.w. The rats were fixed by vascular perfusion through the left ventricle of the heart with 2% v/v glutaraldehyde in 0.05 M sodium phosphate buffer (pH 7.2) for 5 min. After fixation the animals were stored in the same fixative. Following isolation of suitable specimen blocks, the samples were rinsed three times in 0.15 M sodium cacodylate buffer (pH 7.2) and subsequently postfixed in 1% w/v OsO4 in 0.12 M sodium cacodylate buffer (pH 7.2) for 2 h. Furthermore, samples with no osmium post-fixation were included. The specimens were dehydrated in graded series of ethanol, transferred to propylene oxide and embedded in Epon according to standard procedures [20]. Sections of approximately 50 to 60 nm thickness were cut with a Reichert-Jung Ultracut E microtome and collected on copper grids with Formvar supporting membranes. Sections were left unstained or stained with uranyl acetate and lead citrate.

An aqueous feces extract was obtained by the following procedure: 2 ml of deionized water were added to 400 mg of wet feces and carefully shaken until a slurry was formed. The slurry was centrifugated for 10 min at 4,700 rpm (4,224 × *g*) in a Sigma 3-18 K centrifuge (Sigma, Germany) to remove coarse feces constituents. Subsequently 700 μl of the supernatant were transferred to Eppendorf tubes and further centrifugated for 10 min at 13,400 rpm (12,100 × *g*) in a micro centrifuge (Eppendorf MiniSpin, Eppendorf AG, Hamburg, Germany). For TEM analysis 200 μl of the supernatant were taken and diluted two times with deionized water.

The samples were examined with a Philips CM 100 TEM (Philips, Eindhoven, The Netherlands) operated at an accelerating voltage of 80 kV and equipped with a SIS MegaView2 or an OSIS Veleta digital slow scan 2 k × 2 k CCD camera. Digital images were recorded with the analySIS or ITEM software packages and further processed using Adobe Photoshop software (Adobe Systems Incorporated, San Jose, California, CA, USA) to enhance contrast and brightness.

Analysis of the elemental composition was performed by use of a Tecnai T20 TEM (FEI, Eindhoven, The Netherlands) operated at 120 kV and equipped with an INCA energy dispersive x-ray spectrometer (Oxford instruments, Oxfordshire, UK). Analyses were performed with a focused electron probe with an accelerating voltage of 120 kV, a 14° tilt, and a counting time of 100 s. For high resolution imaging an accelerating voltage of 200 kV was applied.

Dried feces was analyzed by a Philips XL 30 FEG scanning electron microscope (FEI, Eindhoven, Netherlands) operated in the backscattered-electron mode at an accelerating voltage of 20 kV.

## Results and discussion

### Characterization and stability study of the silver nanoparticle suspension

**Figure 1:**
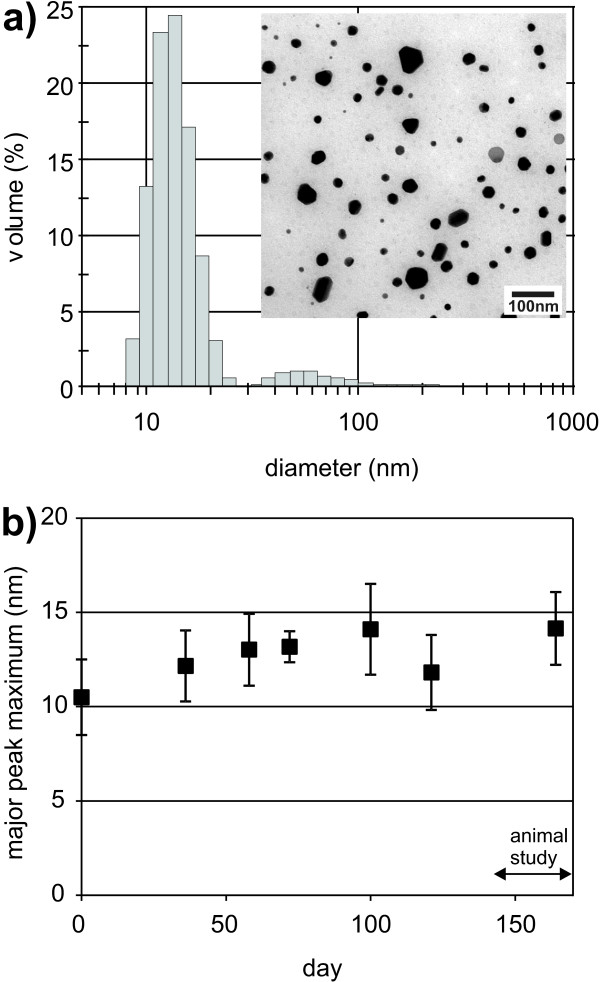
**Size characterization and stability study of the silver nanoparticle suspension**. a) Volume size distribution of the silver nanoparticles determined by dynamic light scattering on day 23 of the 28-day animal study and transmission electron micrograph of the silver nanoparticle suspension, b) Diameter value of the major peak (peak maximum; mean ± 1s.d., N = 5) of the size distribution recorded before and during the animal study.

The size distribution of the AgNPs was determined by DLS (Figure 1a). During the time from the production of the AgNPs until the termination of the animal study the stability of the suspension was monitored by DLS to assure that the NPs were well dispersed during the 28 days duration of the animal experiment (Figure 1b). The volume distribution of the hydrodynamic size of the nanoparticles showed two peaks. The first peak (approximately 90% of the particle volume) had its maximum at 14 ± 2 nm with a peak width of 4 ± 1 nm (Figure 1a), whereas the second peak had its maximum at 50 ± 9 nm with a peak width of 42 ± 3 nm (N = 5). The second peak represents approximately 11% of the total volume of nanoparticles, but less than 0.1% of the total particle number.

TEM was used as a second technique to confirm the nanoparticle sizes obtained by DLS and to obtain information about the particle shape. The nanoparticle diameters observed by TEM were in agreement with the DLS results, but no quantitative analysis was performed with the former technique. As seen in the transmission electron micrograph, (Figure 1a) the nanoparticles had an almost spherical shape and the observed larger size fraction consisted of distinct nanoparticles, and no aggregates of AgNPs were detected.

The zeta potential of the nanoparticles was around -2 mV at a suspension pH of 5.9. Electrostatic stabilization of NPs would typically require a zeta potential above 30 mV or below -30 mV [21]. Accordingly, the stability of the AgNP suspension was solely based on steric stabilization by the PVP polymer. In contrast to electrostatic stabilization, steric stabilization with polymers or proteins is independent of pH and electrolyte concentration. Accordingly, steric stabilization is useful for prevention of agglomeration of nanoparticles in physiological media [21].

The total silver concentration in the suspensions as determined by ICP-MS of microwave-assisted nitric acid digested samples was 628 ± 55 μg/ml (N = 16), which was higher than the nominal concentration of 450 μg/ml. Rats were dosed twice a day with 10 ml/kg b.w. of the AgNP suspension, resulting in a daily silver dose of 12.6 mg/kg b.w. Because the dose of silver acetate was calculated based on the nominal concentration of the AgNP suspension (450 μg/ml), the dose of silver as AgAc at 9 mg/kg b.w. was less than that of the AgNPs.

Most research dealing with the toxicity of silver nanoparticles ignores the possible presence of ionic silver in the nanoparticle suspension. However, we aimed to quantify the proportion of silver in the AgNP suspension not present as nanoparticles. Separation of the AgNPs from the suspension by centrifugation through 12.5 kDa cut-off filters showed that 70 ± 1 μg/ml (N = 3) was present in the filtrate, corresponding to 11% of the total silver concentration. Ultracentrifugation was used to verify the results from the ultrafiltration experiment and to rule out the possibility that silver adhered to the ultrafiltration membrane. The silver concentration in the supernatant from the centrifugation was 65 μg/ml, which confirmed the result of the ultrafiltration experiment. Consequently, the dose per animal of this fraction of silver was approximately 1 mg Ag/kg b.w./day, and remained stable over a time period of at least nine months. The observation that 11% of the total silver concentration was not present as nanoparticles despite purification of the AgNP suspension after synthesis was in agreement with a former study [22]. This study showed by use of a dialysis tube that PVP-coated silver nanoparticles in 5°C water released 10% of the silver in ionic form within approximately 25 days. No further dissolution was observed during the monitored time period of approximately 60 days. In the present study the first filtration experiments were performed one month after synthesis. The suspensions were stored at 4°C throughout the duration of the study. Therefore, the detected silver after filtration and ultracentrifugation could be ionic silver which was released after the synthesis of the AgNPs.

The results demonstrated that a well-characterized nanoparticle suspension was available and dosed to the rats throughout the study. The finding of a fraction of non-nanoparticulate silver in the AgNP suspension provided input to a more detailed interpretation of the findings from the animal study.

### Organ distribution of silver

**Figure 2:**
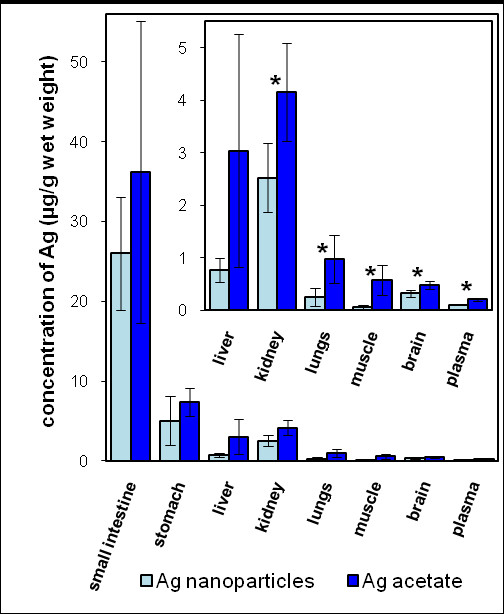
**Silver concentration in rat organs**. Silver concentrations (mean ± 1s.d., N = 5-7) in rat organs after 28 days oral administration of silver nanoparticles and silver acetate. Statistically significant differences (p < 0.05) between the groups are marked with asterisks (*). Silver concentrations in the control animals were below the limit of detection.

Figure 2 presents the silver organ concentration in AgNP and AgAc exposed rats. The silver concentration in organs of the control animals was below the limit of detection (2 ng/g wet tissue). In general, the organ distribution pattern of silver was similar for the two silver dosage forms, AgNPs and AgAc. The highest silver concentrations, listed in decreasing order, were found in small intestine, stomach, kidney, and liver tissues of the exposed animals. However, despite a higher oral silver exposure to the AgNP group the resulting silver concentrations in kidney, lung, muscle, brain, and plasma were lower (p < 0.05) in comparison with the corresponding tissues from animals exposed to AgAc. When normalized to the doses of 12.6 and 9 mg Ag/kg b.w./day used for the AgNP and the AgAc groups (see additional file 1), respectively, the silver concentrations in tissues from animals exposed to AgNPs were approximately 40-50% of those corresponding to administration of AgAc for kidney, stomach, brain and plasma, and 10-20% for muscle and lungs.

This is the first direct comparison of the distribution and concentration of silver in rat organs following oral administration of AgNPs or a soluble silver salt. The distribution of silver following AgAc exposure in the present study resembled that reported in other studies where rats were orally exposed to silver chloride or silver nitrate. Silver was found to be distributed to several tissues, including liver, spleen, bone marrow, lymph nodes, skin, kidney, thyroid, heart, pancreas, adrenal glands, and brain [9]. Furthermore, the silver organ distribution pattern and concentrations of silver following exposure to AgNPs in the present study were comparable to the results from a 28-day study where 30 mg/kg b.w./day AgNPs of 60 nm in diameter and stabilized with carboxymethylcellulose were administered orally to rats [13]. The fact that similar silver concentrations were found in the same organs in both studies was surprising because the dose in the published study [13] was twice as high as that used in the present study. This observation could be explained by a lower absorption rate of silver from the five times larger carboxymethylcellulose-stabilized AgNPs [13]. Furthermore, an influence of the different stabilizing agents on the absorption of the nanoparticles is possible [12].

The relatively large standard deviation for silver in small intestine and liver tissues (Figure 2) was caused by inter-animal variation. Such variation could indicate that despite the same dose per kg body weight, variations in factors such as the intestinal flora and passage time may have an influence on how much silver is actually taken up.

Since 11% of the total silver in the AgNP suspension was present in another form than nanoparticles, it was possible to consider that only this fraction was absorbed by the intestine. The non-nanoparticulate form of silver in the AgNP suspension correspond to a dose of 1 mg Ag/kg b.w./day in comparison to 9 mg Ag/kg b.w./day for AgAc. Assuming similar rates of absorption, accumulation, and excretion, the expected silver concentration would in this case be nine times lower in the blood plasma and organs in comparison to blood plasma and organs from AgAc dosed animals. However, our findings demonstrated that the silver concentrations in stomach, kidney, brain, and plasma of the AgNP exposed rats were approximately one half of that found after administration of AgAc. Therefore, the silver concentration in tissues cannot be ascribed only to the non-nanoparticulate fraction of the AgNP suspensions, but must partially contain silver originating from the AgNPs.

### Excretion of silver

Table 1 presents the absolute and relative amounts of silver in feces and urine after 24 hours of collection in the third week of the study. The excretion is given in both micrograms and percent of the total daily intake. The excretion of silver with urine was low (<0.1%) whereas a high amount of silver was excreted in feces. This finding was in agreement with previous reports on excretion of silver nitrate or silver iodide after oral or intravenous administration to rats [9,23].

**Table 1:** 24 h excretion of silver in urine and feces

	urine (μg)	feces (μg)	urine (% of 24 h intake)	feces (% of 24 h intake)
**Ag-PVP nanoparticles**	0.10 ± 0.05	1190 ± 430	0.005 ± 0.003	63 ± 23

**Ag acetate**	0.73 ± 0.23	610 ± 250	0.057 ± 0.017	49 ± 21

Absolute and relative amount of silver (mean ± 1s.d., N = 5) excreted in urine and feces within a 24 hour time period in week 3 of the study.

The excretion of silver in feces was 63 ± 23% (N = 5) of the daily dose for AgNPs and 49 ± 21% (N = 5) of the daily dose for AgAc. However, given the experimental design it was not possible to provide information as to what extent silver was absorbed from the digestive tract because fecal silver may partially be a result of biliary excretion. In studies using intravenous dosing it was shown that biliary excretion of silver was high [23] and against a plasma-to-bile concentration gradient where the concentration in bile was 16 to 20 times higher than that in plasma from rats [24]. Furthermore, binding of silver to the intestinal surface could result in a decreased excretion of silver within the 24-hour collection of feces.

The non-significant difference between the fecal excretion of silver in the AgNP and AgAc groups suggested a higher fecal excretion of silver after oral exposure to nanoparticles. This was in accordance with the generally lower values for silver in organs of rats that received AgNPs (Figure 2). A possible explanation for a decreased absorption of AgNPs would be an enhanced binding of nanoparticles to non-digestible food components. Furthermore, the passage of the intestinal barrier of AgNPs could be inferior to that of AgAc.

Preliminary investigations of the presence of AgNPs in dried feces by SEM and of an aqueous feces extract by TEM, however, provided no evidence for the presence of AgNPs. Further work in a targeted study is therefore warranted, and should include optimization of the sample preparation methodology and examination of a larger number of samples before any definite statement about the presence or absence of intact AgNPs in the rat feces can be made.

### Localization of silver in the ileum

**Figure 3:**
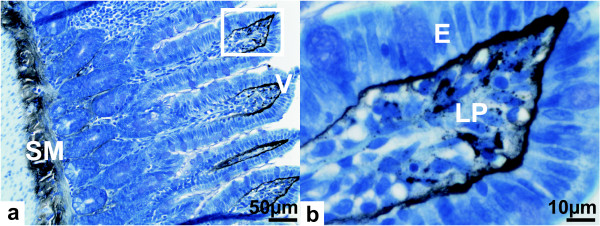
****Autometallographic staining of ileum from a silver nanoparticle exposed rat****. Optical micrographs of ileum section cuts showing, a) Overview and b) Detail with heavy staining in the tips of the intestinal villi (V) directly below the epithelium (E), within the lamina propria (LP), and in the submucosal region (SM).

Autometallographic staining (AMG) was used to analyze the *in situ *distribution of silver in the ileum tissue. In the ileum AMG revealed the presence of silver in the lamina propria mainly in the tips of the villi, whereas no silver was detected in the epithelial cell cytoplasm (Figures 3a and 3b). Furthermore, silver was present in cells in the submucosa. Sections of AgAc exposed rats showed the same distribution of silver in the ileum as observed for AgNP exposed rats (not shown). However, it must be kept in mind that AMG cannot distinguish between silver in the form of nanoparticles, metallic silver, or silver selenide and silver sulfide nanocrystals [19]. Tissue from the control animals showed no staining except for a faint non-specific background which proved that no unspecific staining occurred.

**Figure 4:**
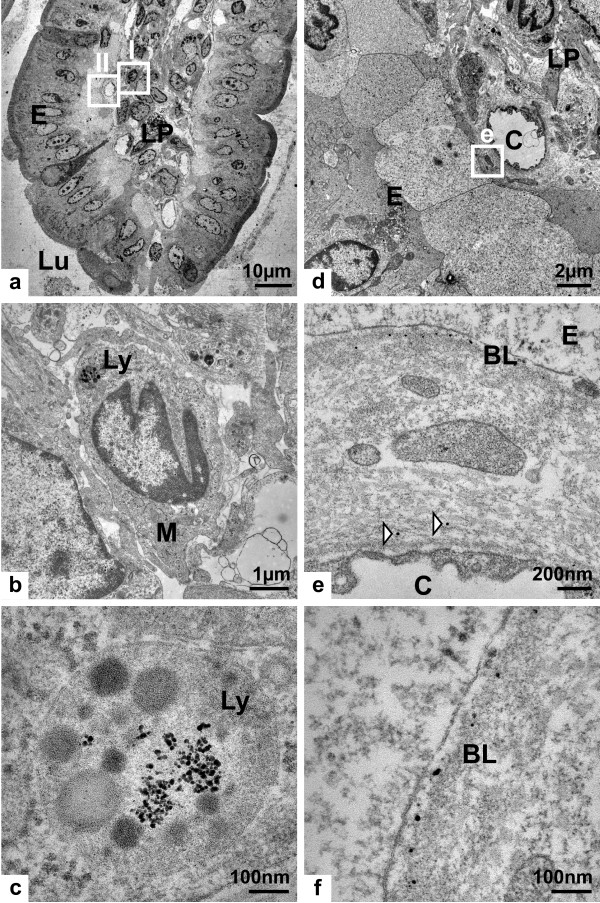
****Transmission electron micrograph of an intestinal villus (ileum) from a silver nanoparticle exposed rat****. Transmission electron micrographs of ultra-thin sections (stained with uranyl acetate and lead citrate) showing a) overview of an intestinal villus (LU = lumen) highlighting two regions of interest (I and II) in the lamina propria (LP) as well as in a region between lamina propria and the epithelium (E), respectively; b-c) details of region I showing granules in a lysosome (Ly) within a macrophage (M); d-f) details of region II showing granules in close proximity to a capillary (C) within the lamina propria and in the basal lamina (BL) of the epithelium. The region of interest highlighted in d) is presented in e), whereas f) is showing the basal lamina in more detail.

**Figure 5:**
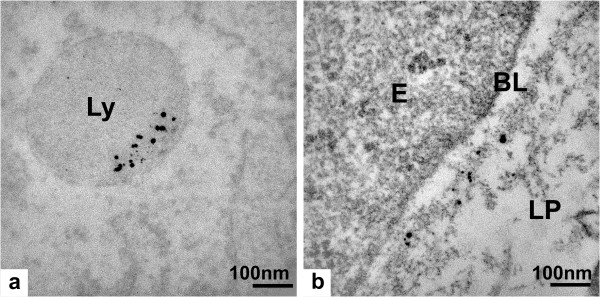
****Transmission electron micrograph of an intestinal villus (ileum) from a silver acetate exposed rat****. Transmission electron micrograph of unstained ultra-thin sections showing granules a) in a lysosome (Ly) within a macrophage in the lamina propria and b) in the basal lamina (BL) of the epithelium.

TEM was used to further characterize the silver accumulated in the intestinal tissue. In the AgNP exposed rats, mostly spherical, but strongly aggregated electron-dense granules were found in the lysosomes of macrophages within the lamina propria (Figures 4a-c) and individual granules in the basal lamina of the epithelium (Figures 4d-f). In the AgAc exposed rats granules of similar size were found at the same positions within the tissue (Figure 5a and 5b). The diameter of the granules was approximately 12 nm or smaller, which was in the same size range as the majority of the administered AgNPs (14 ± 4 nm). Furthermore, single granules were found randomly distributed in the connective tissue of the submucosa. No granules were observed in the microvilli or within the enterocytes, which could be due to the fact that the last dose was administered on the day before the rats were sacrificed. The investigation of unstained samples also showed electron dense granules at the different locations. Thus, artifacts caused by heavy metal staining with lead citrate and uranyl acetate could be excluded.

**Figure 6:**
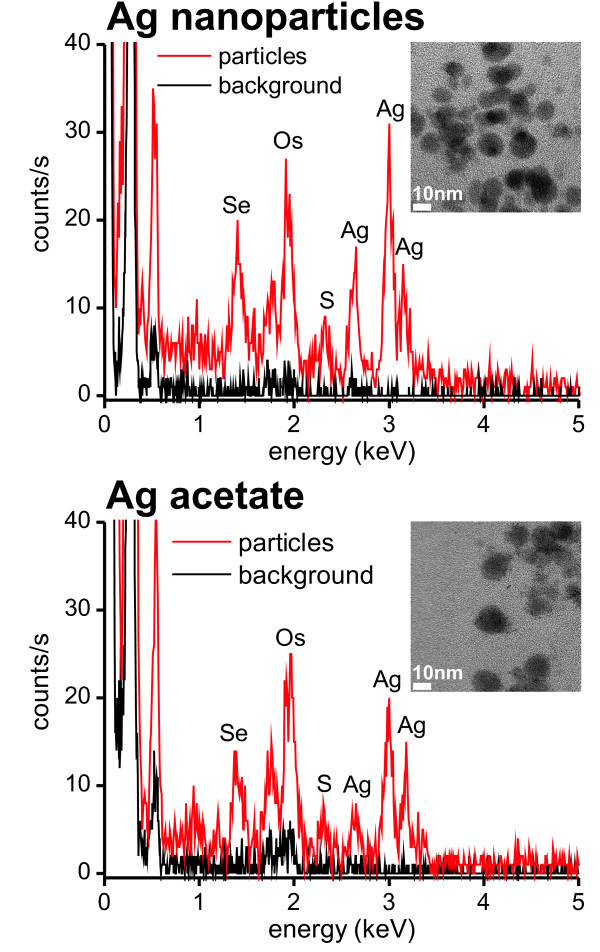
****Energy dispersive x-ray emission spectra of granules in the ileum****. Energy dispersive x-ray emission spectra of granules in lysosomes of silver nanoparticle and silver acetate exposed rats (red line) and corresponding background spectra (black line), *i.e*. spectra of surrounding tissue containing no visible granules (unstained sections). The transmission electron micrographs show the analyzed granule containing area.

Energy dispersive x-ray spectroscopy (EDX) was used to confirm that the observed granules in the lysosomes of macrophages indeed consisted of silver (Figure 6). In addition to silver signals corresponding to selenium and sulfur were detected. These were absent in the background spectrum which was recorded from tissue containing no granules. An osmium signal originated from the tissue fixation procedure in which osmium tetroxide was used as a post-fixative. There was no qualitative difference between the elemental composition of the granules found in the AgNP and AgAc exposed rats.

The observation of sulfur or selenium containing silver granules in the tissue after dosage of AgAc was in agreement with results from other studies. Occupational exposure of humans to silver [25] or intake of silver-containing drugs [26,27] was found to lead to a slate blue-gray irreversible discoloration of the skin of patients termed "argyria". In the skin, granules of typically 20 to 100 nm in diameter were observed which consisted of silver sulfide and silver selenide [26]. In rat studies, 30 to 90 nm granules were observed in the basal lamina of the renal glomeruli after administration of 0.15% aqueous silver nitrate solution in drinking water for 4 to 15 weeks [28,29]. The proton induced X-ray emission microanalysis of silver grains enhanced by autometallography and isolated from kidney sections of silver exposed rats showed that the granules consisted of silver and sulfur [19]. After exposing rats to 0.01% silver lactate or silver nitrate dissolved in drinking water for four weeks, AMG developed silver grains were found in neurons and glia calls, both intracellularly in lysosomes and extracellularly in basement membranes [30]. Furthermore, it has very recently been shown that silver ions are liberated *in vivo *from silver surfaces [31]. AMG developed silver grains were detected both extra- and intracellularly in tissues far away from the implant including regional lymph nodes, liver, kidney, and central nervous system [31]. However, the formation of selenium and sulfur containing silver granules both in the intestinal system of rats after oral exposure to AgAc or to AgNPs has not been described so far.

The presence of sulfur in the *in vivo *formed silver granules has been explained with the high affinity of silver and sulfur which could result in a combination of silver ions with biological structures and constituents with high sulfur content. Such examples include cystein contained in collagen fibers or sulfur-containing polyanionic glycoproteins which are particularly abundant in the basal lamina [19,26]. This would be in accordance with the observed deposition of granules in the basal lamina of the ileal epithelium in the present study (Figure 4f). The lysosomal accumulation of silver could be caused either by direct uptake from the external milieu such as protein-bound silver or by primary binding of silver to intracellular molecules which were subsequently engulfed and digested [30]. The presence of selenium in the silver granules has been explained by a possible replacement of sulfur by selenium over time because of the higher chemical stability and lower solubility of silver selenide in comparison to silver sulfide [26,32]. Another suggested mechanism has been the direct interaction of silver with selenium bound to glutathione peroxidase (GSH-Px) [26]. The formation of the insoluble silver sulfide and selenide reduces the toxic effects of silver ions by reducing their biological availability and consequently preventing their interference with normal enzymatic activities in tissues [26,32].

The present study showed the formation of silver granules containing selenium and sulfur in lysosomes following administration of AgNPs. We suggest two possible scenarios: 1) AgNPs were dissolved in the gastrointestinal system. The silver ions bound to proteins and were deposited as granules in the lysosomes in a similar way as silver acetate. 2) Intact AgNPs were absorbed, bound to proteins and subsequently deposited in lysosomes. In the first scenario, it would be a coincidence that the re-deposited silver formed granules with a similar size as the administered silver nanoparticles. In the latter case, in would be a coincidence that the granules deposited after administration of silver acetate had a similar size as the deposited silver nanoparticles.

It is possible that the nanoparticles are taken up across the intestinal barrier as particles with diameters less than 1 μm are extremely susceptible to absorption by the intestinal system [11]. Gold nanoparticles with diameters ranging from 5 to 58 nm were administered orally to mice. It was shown that the gastrointestinal absorption of the nanoparticles occurred paracellularly by persorption through gaps created by extruding enterocytes [33]. However, the question is whether the AgNPs remain intact in the gastrointestinal system prior to absorption.

The size distribution (Figure 1a) showed that the AgNP suspension contained a small fraction of nanoparticles equal to or larger than 50 nm in diameter. In contrast, nanoparticles of this size fraction were not observed in ileum by TEM despite the expected high degree of absorption of nanoparticles in this size range [34]. This suggests at least a partial dissolution of these large particles in the gastrointestinal system. Furthermore, other studies have shown that polyvinylpyrrolidone (PVP)-coated AgNPs reacted with hydrochloric acid (18%) to form insoluble AgCl, which did not occur for bulk or coarse-grained silver [35], and that AgNPs completely dissolved in aqueous nitric acid solution at pH = 0.5 [36]. Although these experiments were not conducted under physiological conditions, they indicate that AgNPs may undergo chemical reactions and eventually dissolve in the gastrointestinal system. Further dedicated studies that focus on the stability of AgNPs in the gastrointestinal environment are therefore warranted.

### Localization of silver in the liver

Using AMG, silver could also be localized in the liver tissue of the exposed rats. In the tissue from the untreated control animals no staining, except for a faint non-specific background in some sections, was observed. The large inter-animal differences in the silver concentration in the liver observed by ICP-MS analysis were confirmed by the AMG stained sections in which differences in the staining intensity of the liver sections from different animals were observed, though similar parts of the livers were investigated. The distribution pattern of silver was again similar for AgNP and AgAc exposed animals. In general an intense staining was observed around the central veins and the portal tracts (see additional file 2a). In these regions several Kupffer cells were intensely stained. However, there was no preferred staining of Kupffer cells in the remaining tissue. Small AMG grains were mainly observed in or around hepatocytes (see additional file 2b). Whether these AMG grains were present within the hepatocytes or in the bile canaliculi could not be determined. The observations were in contrast to findings for intravenously and intraperitoneally injected gold nanoparticles which, independent of their size, were taken up primarily by the Kupffer cells in the liver [37] and could indicate that no intact silver nanoparticles were deposited in the liver. No silver granules could be detected by TEM, which could either be related to a very small size or a low number of the granules in the tissue. A few silver atoms are sufficient to be visible as AMG grains after staining [19], which might not be detectable by TEM.

### Localization of silver in the kidneys

In kidney tissue silver AMG staining revealed the presence of silver in the glomeruli and proximal tubules (see additional file 3a). In the proximal tubules single large AMG grains were localized along the luminal brush border of the epithelium (see additional file 3b), whereas the staining was more homogenous in the glomeruli. Furthermore, heavy staining was observed in the renal papilla and seemed to be restricted to the interstitial tissue (images not presented). Again, no difference in the silver distribution after AMG staining was observed between AgNP and AgAc exposed animals. However, on the basis of AMG staining no statement about the chemical form of the deposited silver can be made.

First investigations of an AgNP exposed rat by TEM revealed the presence of granules in the microvilli of the epithelial cells of a proximale tubule (see additional file 4), which was in agreement with the large AMG grains observed by light microscopy. The granules had an irregular shape and a relatively broad size distribution, which did not coincide with that of the administered AgNPs and could be agglomerates of the nanoparticles. Future investigations will focus on the chemical composition of these granules.

No granules were found in the basal lamina of the renal glomeruli by TEM. In studies which described the deposition of silver in the basal lamina of the renal glomeruli, animals were exposed for several weeks by adding silver to the drinking water with concentrations around 2.5 mg/ml [38]. It was found that in immature rats the deposition of silver took place much more gradually and that the granules were smaller and more sparsely distributed in comparison to adult rats [30]. This could explain the absence of granules visible by TEM in the glomerular membrane in the present study. Lung, muscle, and brain tissue were not analyzed by microscopy because of the relatively low silver concentrations in those tissues.

## Conclusions

In the present study, the comparison of silver acetate and silver nanoparticles did not reveal differences with respect to the distribution pattern of silver in organs and tissues. The absolute silver concentrations in the organs were generally lower after administration of AgNPs than after administration of AgAc. This was in agreement with an indication of a higher fecal excretion of silver after administration of AgNPs, and further studies are warranted.

In the ileum of rats exposed to either AgNPs or AgAc, sulfur and selenium containing silver granules of similar size and shape were found in lysosomes of macrophages. The deposited granules resulting from both tested forms of silver were in the same size range as the administered silver nanoparticles. The coinciding size ranges however, hampered the identification of the absorption mechanism of the nanoparticles. Further studies will be necessary to investigate whether silver nanoparticles are absorbed as an entity or whether they dissolve in the gastrointestinal system followed by re-deposition in the tissues. The accumulation of silver from AgAc and from AgNPs points to lysosomes as a likely target for adverse effects.

In the design of future nanotoxicity studies using oral administration of nanomaterials to laboratory animals, the possible dissolution of the nanoparticulate test material in the gastrointestinal system should be taken into account. Furthermore, inclusion of a non-nanoparticulate counterpart to the tested nanomaterial for comparison of toxicity is advisable.

## Competing interests

The authors declare that they have no competing interests.

## Authors' contributions

KL carried out the silver organ distribution study, the light and transmission electron microscopy analysis, dynamic light scattering measurements, and drafted the manuscript. NH was involved in design and execution of the animal study. KQ was responsible for TEM sample preparation and supported interpretation of the TEM images. AL provided the AMG staining method and revised the manuscript. XG was responsible for preparation of the AgNPs and contributed to the nanoparticle characterization. UV has revised the manuscript. AM was responsible for the design and conduction of the animal study, participated in light microscopy analysis of AMG developed tissue cuts, and revised the manuscript. HRL contributed to the design of the study and revised the manuscript. EHL contributed to design and coordination of the present study and revised the manuscript. All authors read and approved the final manuscript.

## Supplementary Material

Additional file 1 **Normalized silver concentrations in rat organs**. Silver concentrations (N = 5-6) in the rat organs after normalization to the doses of 12.6 and 9 mg Ag/kg b.w./day administered to the AgNP and the AgAc group, respectively, as well as the ratio between mean silver organ concentrations in the AgNP and AgAc groups (marked as dots). Statistically significant difference (p < 0.05) between the groups is marked with asterisks (*). Click here for file

Additional file 2 **Autometallographic (AMG) staining of liver from an AgNP exposed rat**. a) Portal triad with heavy staining in Kupffer cells and around the blood vessels b) AMG grains scattered around hepatocytes Click here for file

Additional file 3 **Autometallographic (AMG) staining of kidney from an AgNP exposed rat**. a) Heavy staining of the glomeruli b) AMG grains in a proximal tubule Click here for file

Additional file 4 **Transmission electron micrograph of a renal proximal tubule from an AgNP exposed rat**. TEM images of ultra-thin sections (stained with uranyl acetate and lead citrate) of kidney a) Overview of a proximal tubule with the region of interest shown in b); b) ensemble of granules in the microvilli of the epithelia cells; c-d) higher magnification of the granules. Click here for file
