# Supplementary figures and images for: Distribution of silver in rats following 28 days of repeated oral exposure to silver nanoparticles or silver acetate
Source: Part Fibre Toxicol. 2011 Jun 1;8:18. doi: 10.1186/1743-8977-8-18 (PMC3123173; doi:10.1186/1743-8977-8-18)

## Slide 1
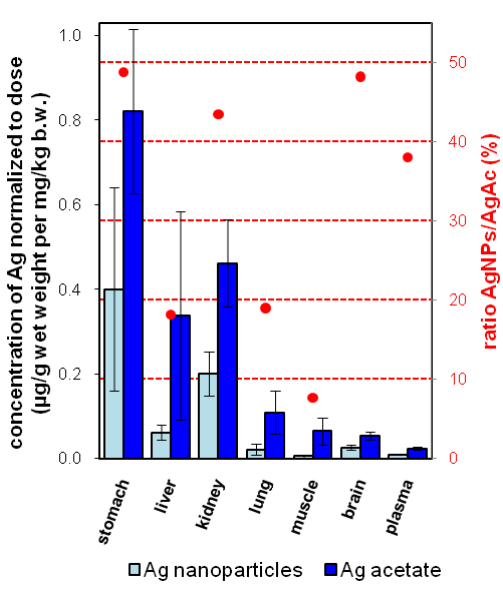

Supplement: Additional file 1 — Normalized silver concentrations in rat organs. Silver concentrations (N = 5-6) in the rat organs after normalization to the doses of 12.6 and 9 mg Ag/kg b.w./day administered to the AgNP and the AgAc group, respectively, as well as the ratio between mean silver organ concentrations in the AgNP and AgAc groups (marked as dots). Statistically significant difference (p < 0.05) between the groups is marked with asterisks (*). [file 1743-8977-8-18-S1.PPT]

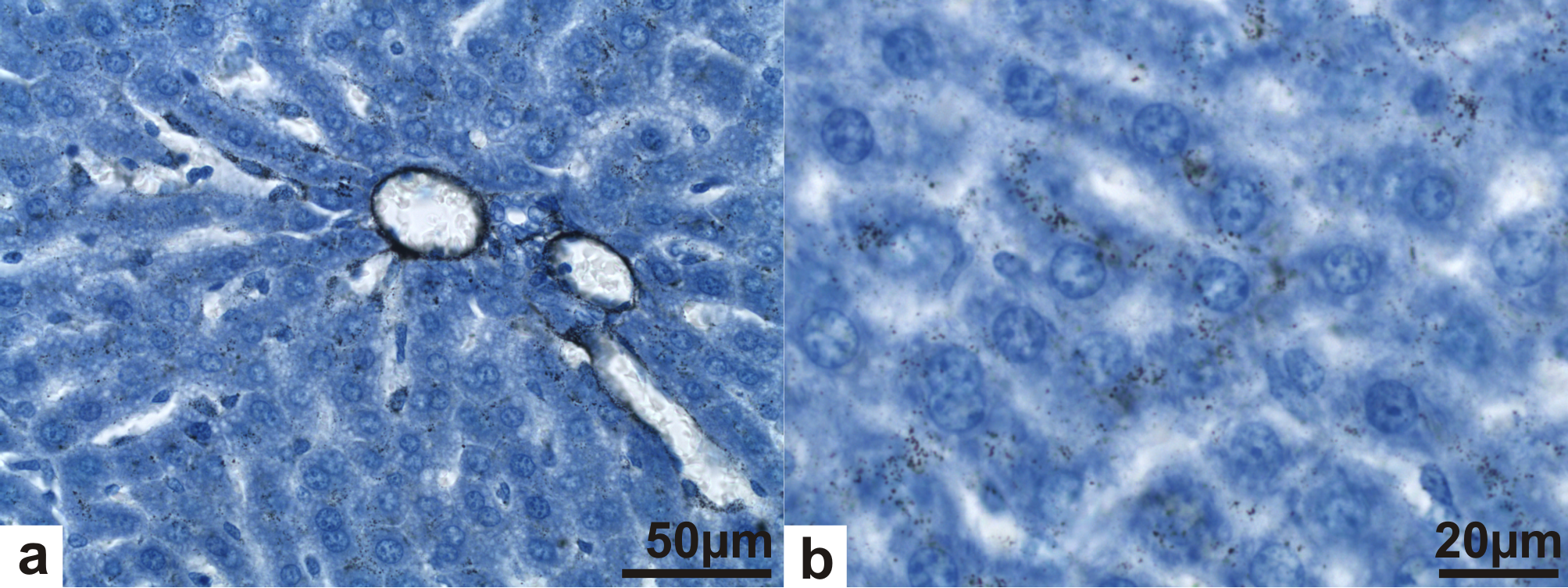

Supplement: Additional file 2 — Autometallographic (AMG) staining of liver from an AgNP exposed rat. a) Portal triad with heavy staining in Kupffer cells and around the blood vessels b) AMG grains scattered around hepatocytes [file 1743-8977-8-18-S2.TIFF]

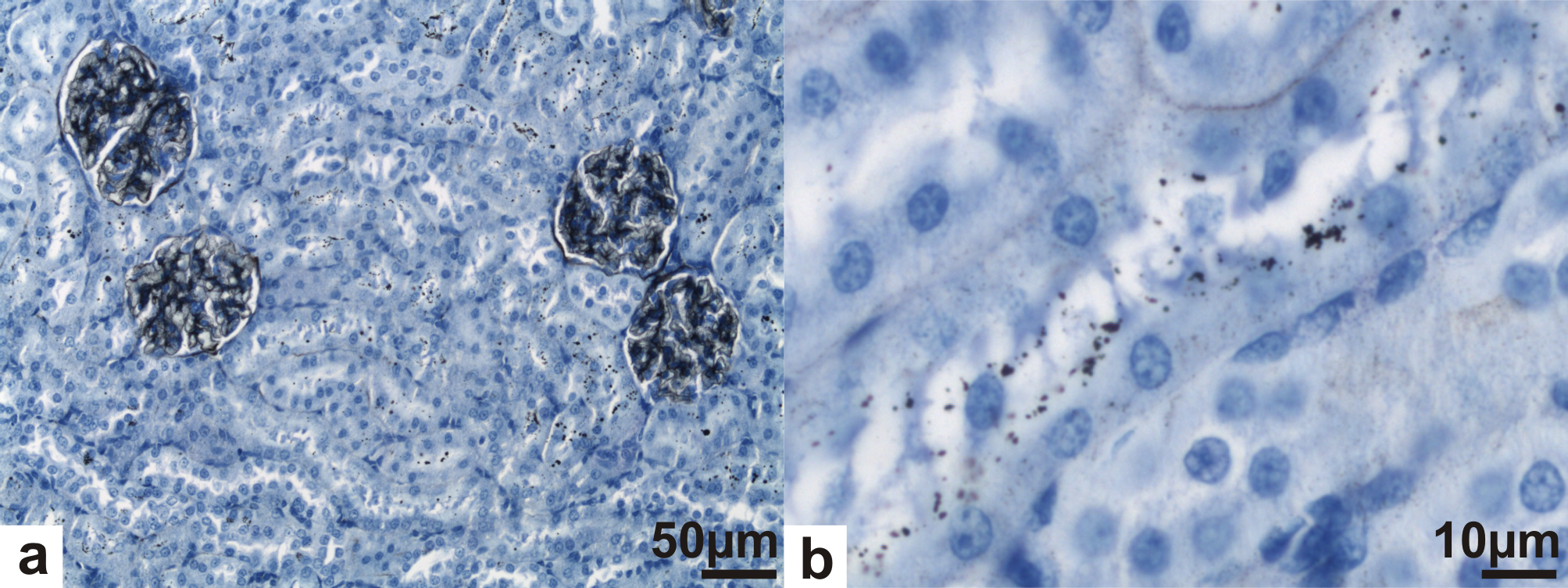

Supplement: Additional file 3 — Autometallographic (AMG) staining of kidney from an AgNP exposed rat. a) Heavy staining of the glomeruli b) AMG grains in a proximal tubule [file 1743-8977-8-18-S3.TIFF]

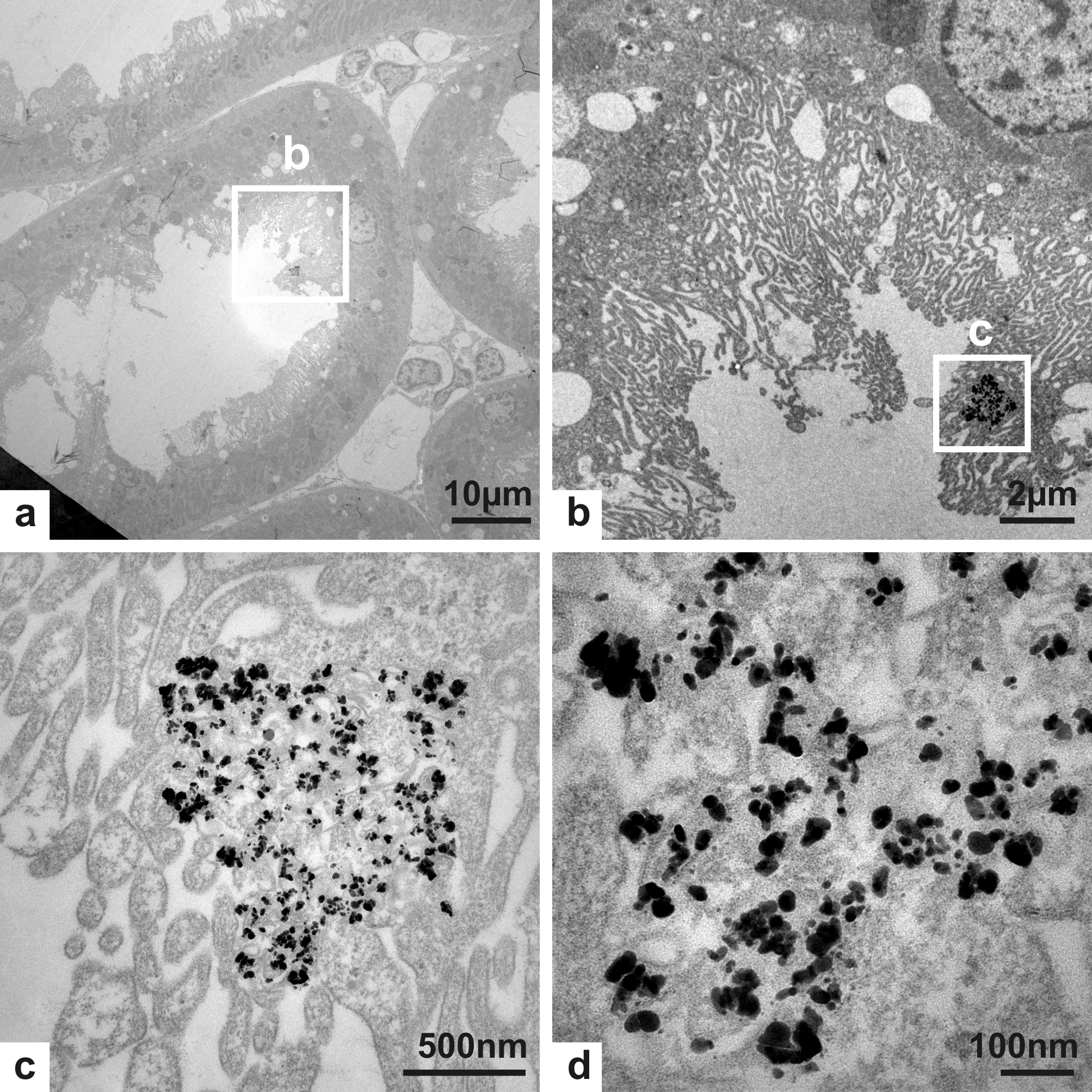

Supplement: Additional file 4 — Transmission electron micrograph of a renal proximal tubule from an AgNP exposed rat. TEM images of ultra-thin sections (stained with uranyl acetate and lead citrate) of kidney a) Overview of a proximal tubule with the region of interest shown in b); b) ensemble of granules in the microvilli of the epithelia cells; c-d) higher magnification of the granules. [file 1743-8977-8-18-S4.TIFF]
